# Supplementary material for: Inflammasome activity is controlled by ZBTB16-dependent SUMOylation of ASC
Source: Nat Commun. 2023 Dec 20;14:8465. doi: 10.1038/s41467-023-43945-1 (PMC10733316; doi:10.1038/s41467-023-43945-1)
Supplement: Supplementary file 1 — Supplementary Information [file 41467_2023_43945_MOESM1_ESM.pdf]

Supplementary figures

a

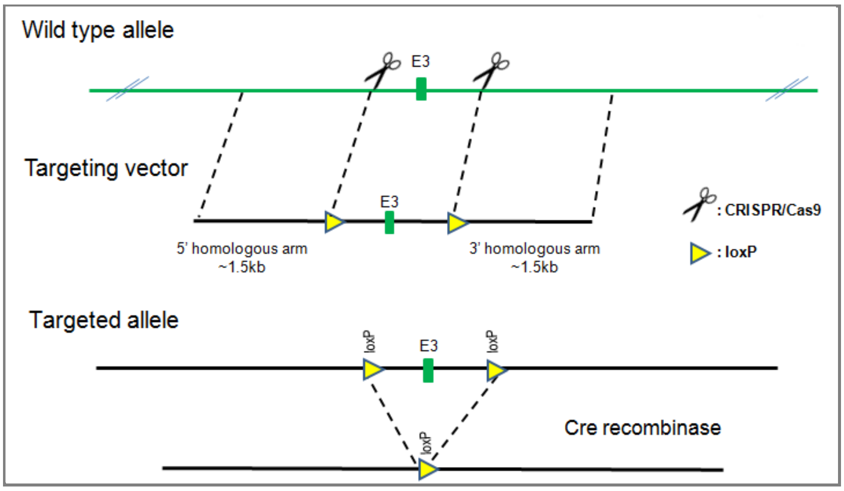

b

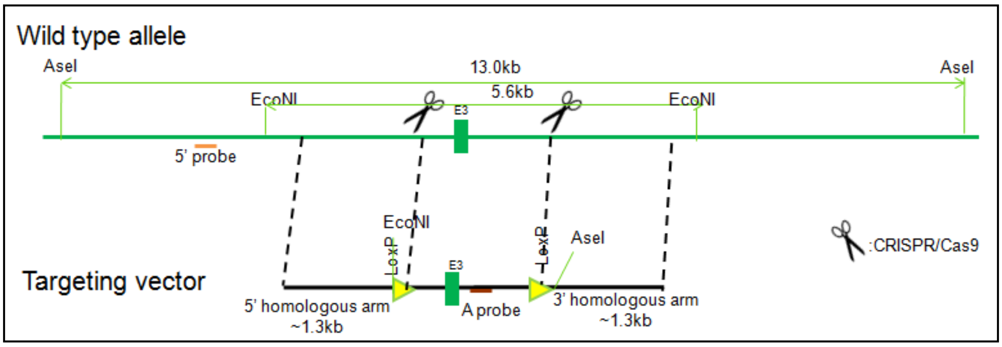

c

Southern blot strategy			
Restriction enzyme	Probe	WT	Targeted
AseI	5' probe	13.0kb	8.2kb
EcoNI	A(3') probe	5.6kb	4.2kb

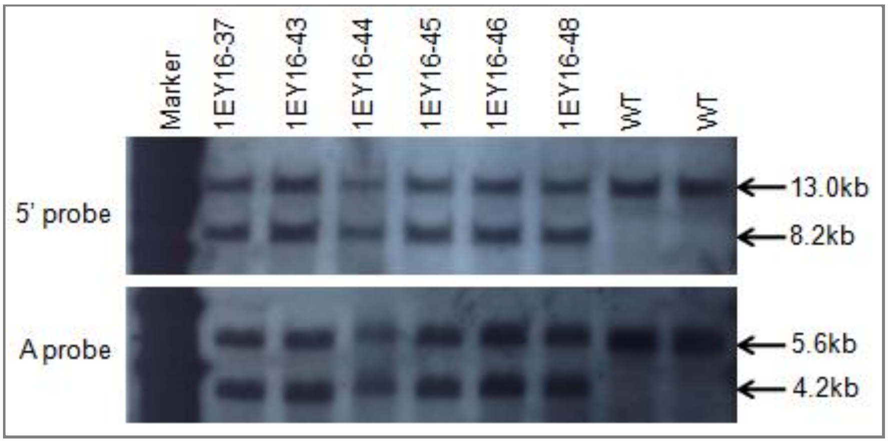

d

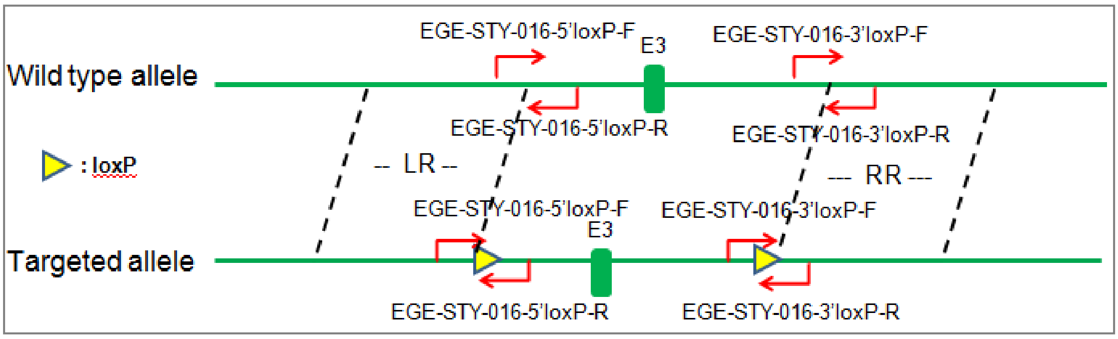

Primer	Sequence (5'-3')	Tm(℃)	Product size
EGE-STY-016–5'loxP-F	CCTTCTGAGTATGGCAGATGGCAGG	65	WT: 333bp<br>Mut: 433bp
EGE-STY-016–5'loxP-R	GACAATCCCCATATGTCCTGTGCCC	65	
EGE-STY-016–3'loxP-F	AGCATGTGATTCCAATCCCCACTCG	65	WT: 339bp<br>Mut: 418bp
EGE-STY-016–3'loxP-R	ACACCATCATCCACAGACAACCCAC	65	

e

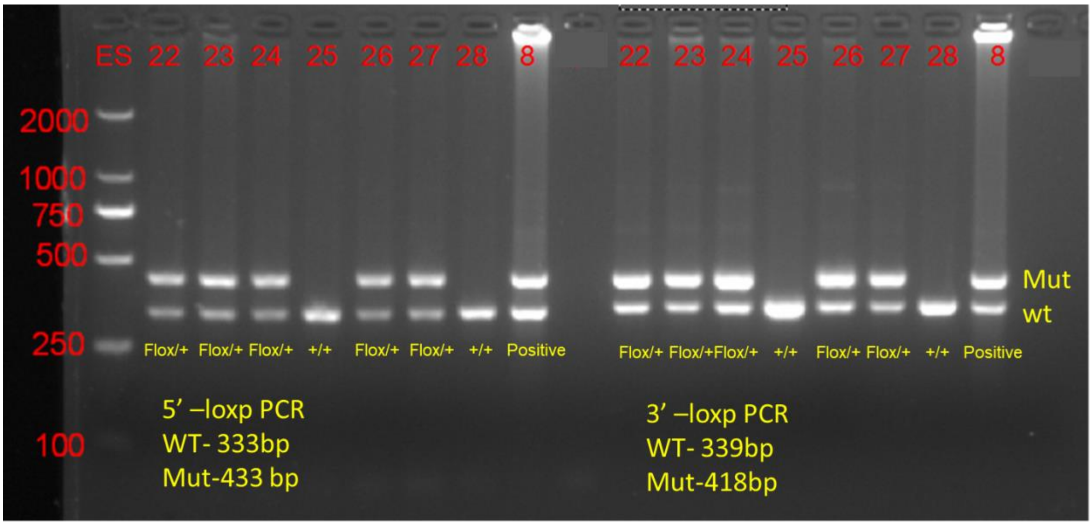

Supplementary Fig 1. Conditional targeted mutagenesis of the *Zbtb16* locus. (**a**) The targeting strategy used to insert a cassette with X-over P1 (*loxP*) sites into the *Zbtb16* locus by CRISPR/Cas9 gene editing that permits subsequent excision of exon 3 of the locus by Cre recombinase. (**b and c**) The screening strategy that was used to identify recombinants by (**b**) restriction endonuclease digestion and hybridization with discriminatory probes (*5'* and *A(3)'*) that were then detected by (**c**) autoradiograph of Southern Blots of targeted clones. (**d**) The genotyping strategy that was used to identify *Zbtb16* (coded as EGE-STY) mutant mice generated from correctly targeted clones by PCR using two primer pairs flanking each *loxP* recombination site. (**e**) Amplicons from mice separated by agarose gel electrophoresis and visualized by fluorescent nucleic

acid staining. The lower molecular weight amplicon from WT animals (samples 25 and 28) is increased by the introduced *loxP* site in mutant mice. Both the WT and mutant amplicons are visible in the heterozygous mice. The *Zbtb16* floxed mice were used as a control ($Zbtb16^{fl/fl}$) for the myeloid cell lineage knockouts that were generated by Cre expression from the lysozyme 2 gene promoter ($Zbtb16^{fl/fl/LysMcre}$) in a model of acute peritonitis induced by intraperitoneal injection of MSU that are shown in Figure 1.

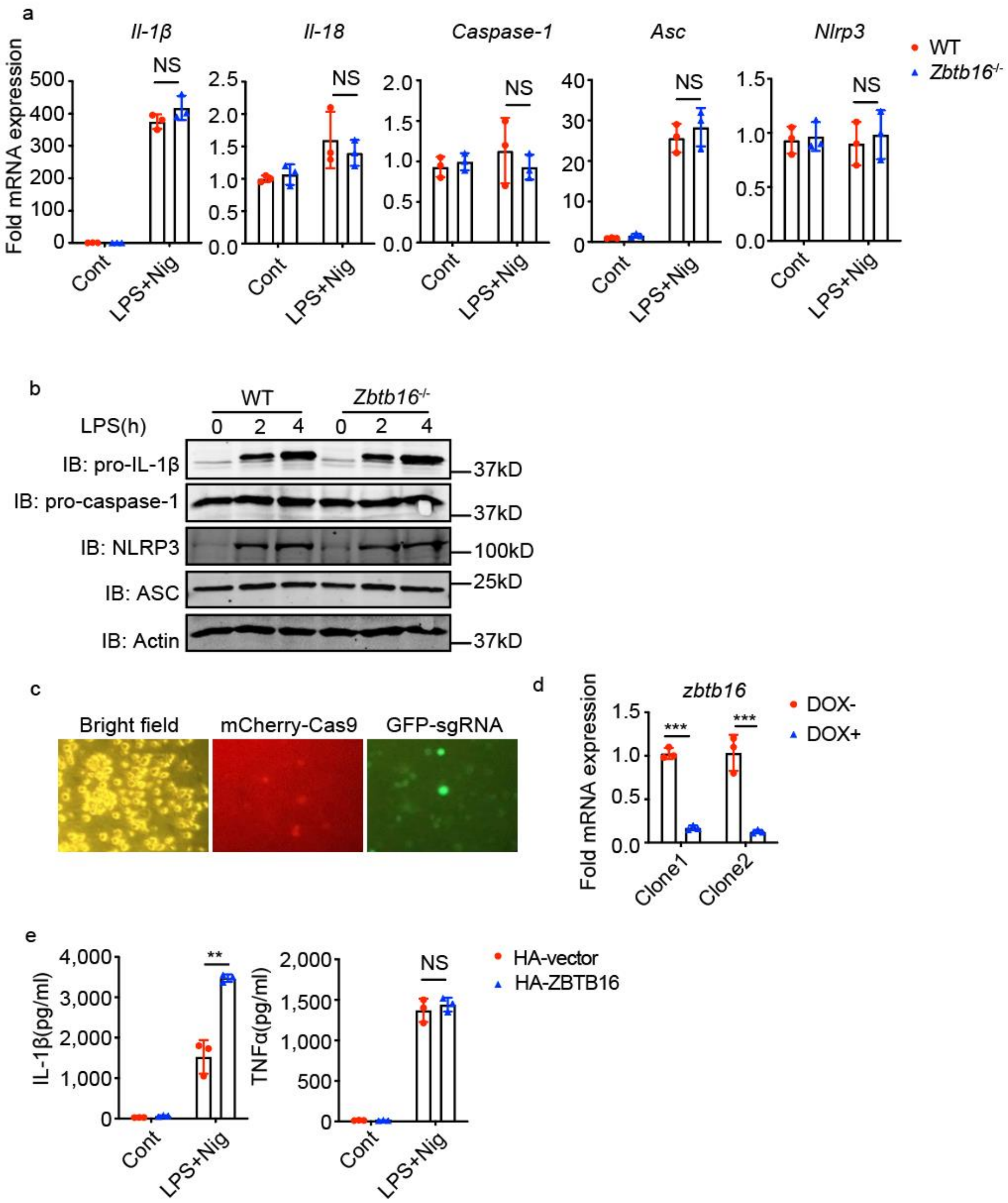

Supplementary Fig 2. The effect of ZBTB16 on the expression and activity of inflammasome constituents. (**a & b**) Measures of the levels of the indicated inflammasome factors in BMDMs from WT and *Zbtb16*$^{-/-}$ mice by (**a**) Q-PCR of cells either left untreated or primed with 1 μg/ml LPS for 4h, followed by stimulation with 20 μM nigericin for 30min and by (**b**) IB of lysates from cells stimulated with 1 μg /ml LPS for the indicated times. (**c-e**) Confirmation of targeting of ZBTB16 expression in the human monocytic cell line THP-1 by CRISPR/Cas9 and DOX-inducible sgRNA by (**c**) visualizing fluorescent reporters in the CRISPR/Cas9-mCherry and *ZBTB16*-sgRNA-GFP constructs in bright field and fluorescent images of THP-1 cells transformed with lentiviral constructs and (**d**) Q-PCR measures of the levels of the *ZBTB16* transcript in two recombinant clones uninduced or induced with 1 μg/ml DOX for four days. (**e**) Measures of the production of the IL-1β and TNFα cytokines by

CRISPR/Cas9+*ZBTB16*-sgRNA THP-1 cells also transduction with control (empty) or ZBTB16 expressing constructs, then either left untreated (Cont) or induced with DOX before stimulated with LPS and nigericin as assayed by ELISA (n=3). Data are presented as mean values ±SD. Statistical significance (*=P<0.05, **=P<0.01, ***=P<0.001) was calculated by a two-tailed Student's *t*-test. The CRISPR/Cas9+*ZBTB16*-sgRNA THP-1 cells were used to generate the data shown in Figure 2. Source data are provided in a Source Data file.

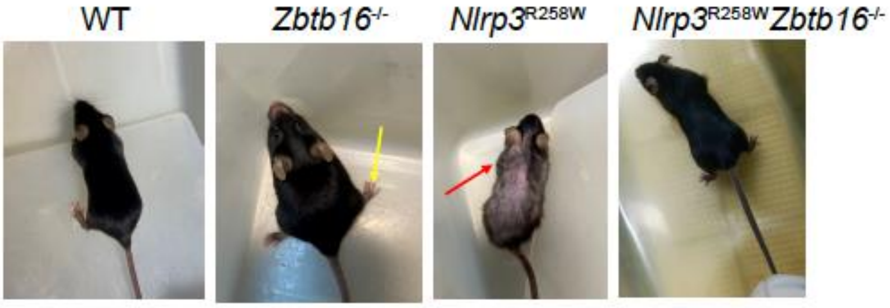

Supplementary Fig 3. The effect of Zbtb16 on a constitutive hyperactive inflammasome. Representative photographs showing the coat phenotypes of the indicated murine lines. Nlrp3^{R258W} mice exhibit spontaneous and contact-induced skin inflammation (indicated with red arrow) that is absent in the WT and *Zbtb16*$^{-/-}$ mice and much reduced in the Nlrp3^{R258W}/*Zbtb16*$^{-/-}$ strain. *Zbtb16*$^{-/-}$ mice commonly exhibited congenital syndactyly as indicated with the yellow arrow. These murine lines were used to generate the data shown in Figure 3.

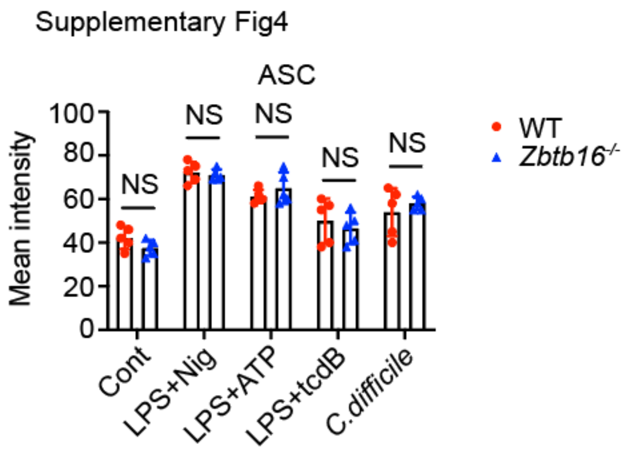

Supplementary Fig 4. Measures of the effect of Zbtb16 on Asc expression. Immunofluorescent detection of Asc in BMDMs from WT and *Zbtb16$^{-/-}$* mice either untreated (Cont) or primed with 1 µg/mL LPS for 4 h, followed by stimulation with 20 µM nigericin or 5 mM ATP for 30 min or 200 ng/mL of TcdB toxin for 4 h or *C. difficile* 630 at MOI 100 for 6 h and then stained with an anti-ASC antibody and quantitated by immunofluorescence (n=5). Data are presented as mean values ±SD. Statistical significance was calculated by a two-tailed Student's *t*-test (NS=not significant). ASC oligomerization and other measures of inflammasome activity induced by these stimuli are shown in Figure 4. Source data are provided in a Source Data file.

Supplementary Fig5

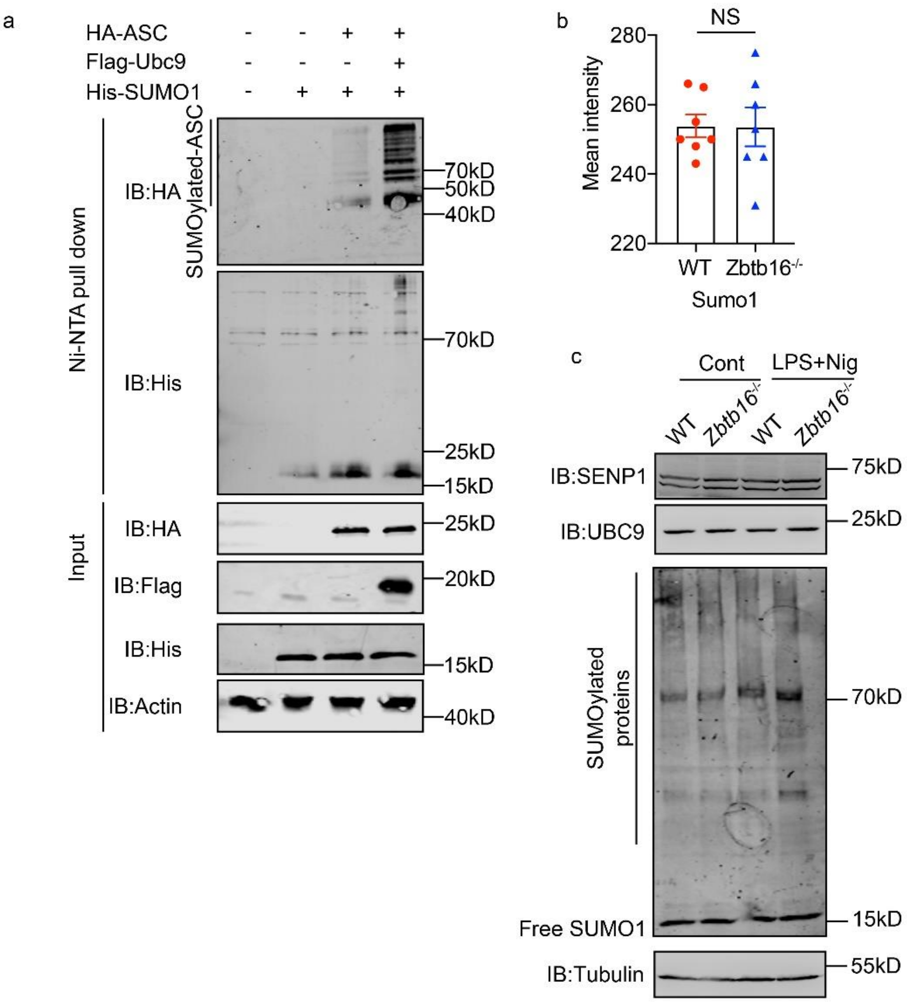

Supplementary Fig 5. Analysis of ASC SUMOylation. **(a)** Detection of the conjugation of SUMO1 to ASC by IB of lysates from HEK-293T cells variably expressing HA-ASC, Flag-UBC9 and His-SUMO1. The recombinant SUMO1 and its conjugates were enriched by purification with Ni-NTA resin and enriched proteins were then detected by IB with the indicated antibodies. **(b)** A comparison of the relative levels of SUMO1 in untreated WT and *Zbtb16*$^{-/-}$ BMDM as detected by immunofluorescence of cells stained with anti-SUMO1 antibody. Fluorescence was quantified using ImageJ software from 5-7 cells per image, in 6-7 random images of each cell line (n=7 per group). Data are presented as mean values ±SD. Statistical significance was calculated by a two-tailed Student's *t*-test (NS=not significant). Source data are provided in a Source Data file. **(c)** Comparisons of the levels of endogenous Ubc9, the SUMO protease SENP1 and as a control Tubulin in WT and *Zbtb16*$^{-/-}$ BMDM at rest (Cont) or upon stimulation with 1 µg/mL LPS for 4 h followed by 20 µM nigericin for 30 min by IB. These data support the measures of ASC SUMOylation that are shown in Figure 5.

Supplementary Fig6

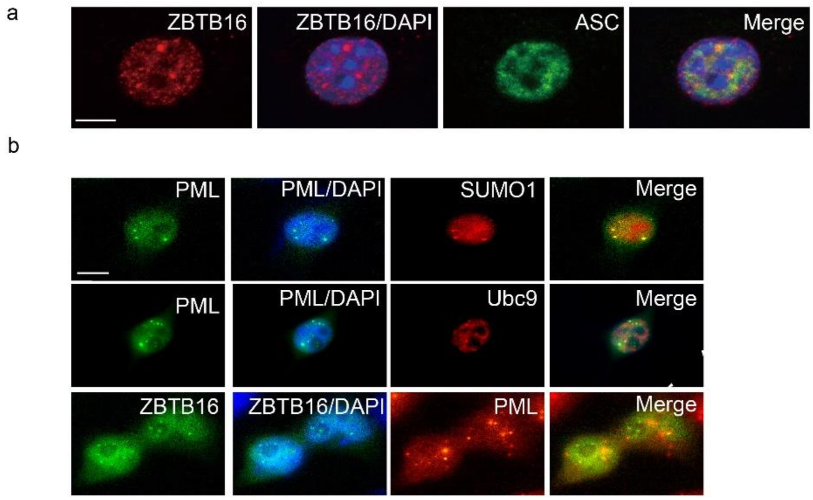

Supplementary Fig 6. Measures of the localization of SUMO factors. **(a)** Micrographs showing the location of ZBTB16 and ASC in BMDMs by immunofluorescence in cells transfecting with ZBTB16 and stained with DAPI (blue), mouse anti-ZBTB16 and rabbit anti-ASC primary antibodies followed by Alexa Fluor 555 anti-mouse (red) and Alexa Fluor 488 anti-rabbit (green) secondary antibodies. Images were acquired by confocal microscopy. Scale bar indicates 10 µm **(b)** Micrographs showing labelling with antibodies against the SUMO E3 enzyme PML, E2 enzyme Ubc9, SUMO1 or Zbtb16 in BMDM to visualize their location in the nucleus, as detected by DAPI staining (blue). Coordinated detection with pairs of antibodies shows the extent of the colocalization of PML and SUMO1, Ubc9 or Zbtb16. Consistent with the PML function requiring its SUMOylation, the patterns of PML and SUMO1 in the nucleus substantially overlap, while the coincidence of the PML and Ubc9 or Zbtb16 signals is less evident (scale bars=10µm). These data support the measures of an association between ASC, SUMO1, UBC9 and ZBTB16 that are shown in Figure 6.

Supplementary Fig7

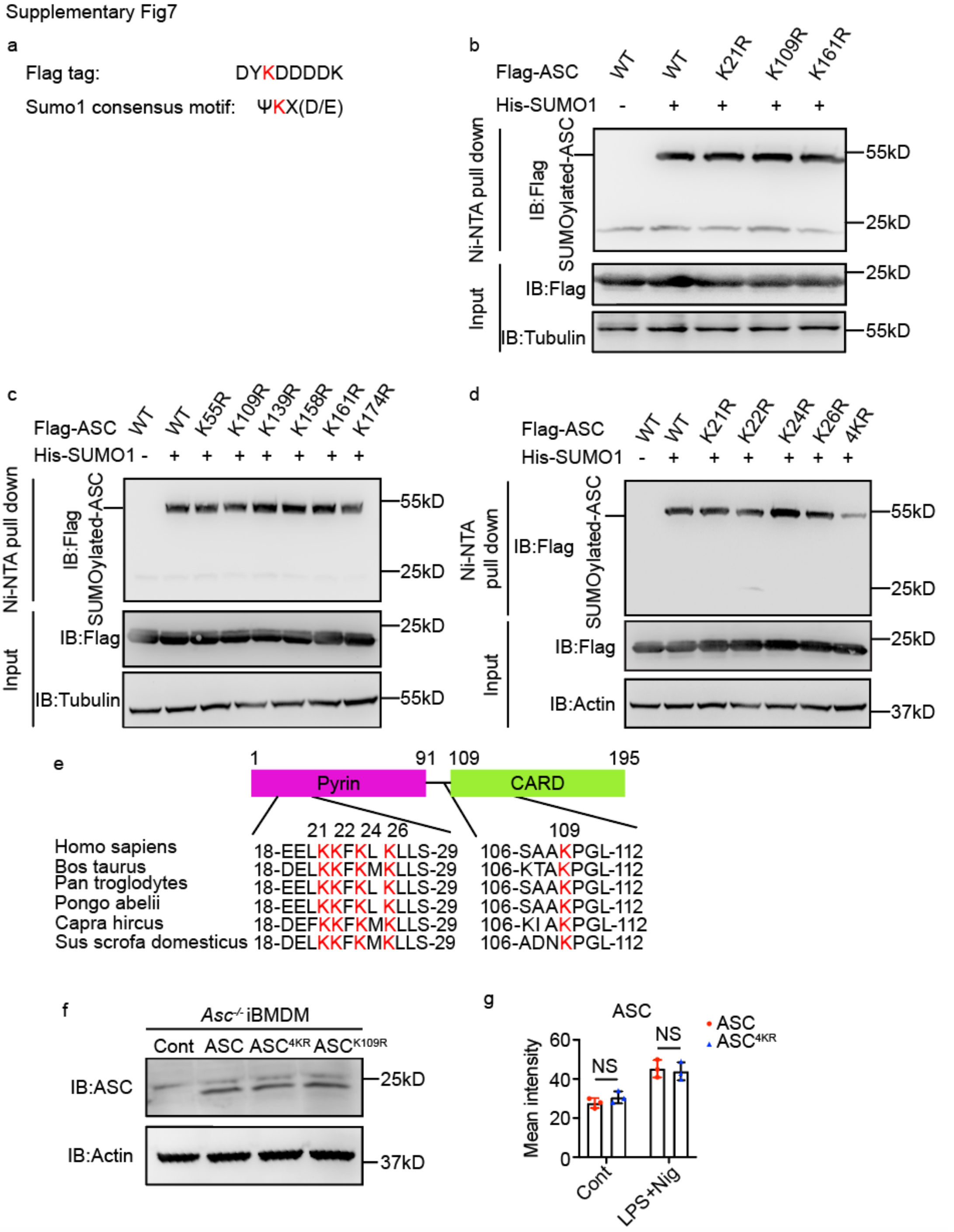

Supplementary Fig 7. Identifying residues on ASC that control its SUMOylation.

(**a**) The amino acid sequence of the Flag epitope in single letter code (at the top) showing it conforms to a SUMO motif (below, Ψ denotes a large hydrophobic residue and X any residue). This suggests that lysine residues in Flag may be SUMOylated when attached to a UBC9 substrate protein. Accordingly, the SUMOylation of Flag-tagged ASC is preserved, as detected by immunoblot (**b & c**), despite separately mutating all lysine residues on ASC, while mutating the lysine residues at positions 21 and 109 reduced the SUMOylation of an HA (YPYDVPDYA) -tagged ASC, as seen in Figure 7. ASC SUMOylation is assessed by nickel enrichment of lysates from HEK-

293T expressing HA-UBC9, His-SUMO1 and Flag-ASC followed by IB with an anti-Flag antibody. **(d)** Combined mutation of the lysine residues between positions 21 to 26 of ASC (4KR), which disrupts interaction with ZBTB16 (as seen in Figure 7), also reduced SUMOylation of Flag-ASC in HEK-293T also expressing HA-UBC9 with His-SUMO1 as assessed by enrichment with Ni-NTA followed by IB with the indicated antibodies. (**e**) A schematic of the PYD and CARD domains of ASC (at the top) indicating the positions of residue numbers 21 and 109 and, in more detail below, an alignment of the amino acid sequence around each residue from the indicated species. Putative ASC SUMO residues conserved between species are shown in red. (**f**) Measures of the relative expression of the WT or the indicated mutant ASC constructs re-expressed in immortalized *Asc*$^{-/-}$ BMDMs (shown in Figure 7) by immunoblotting the lysates with anti-ASC and -Actin antibodies. (**g**) The relative expression of ASC in *Asc*$^{-/-}$ BMDMs reconstituted with either the WT or the 4KR mutant ASC constructs either untreated (Cont) or stimulated with 1 μg/ml LPS for 4h followed by 20 μM nigericin for 30 mins as assessed by immunofluorescence using an anti-ASC antibody (n =3). Data are presented as mean values ±SEM. Statistical significance was calculated by a two-tailed Student's *t*-test (NS=not significant). Source data are provided in a Source Data file. These data support the identification of lysine residues that were demonstrated to regulate ASC SUMOylation and oligomerization which are shown in Figure 7.

Supplementary Fig 8. Full immunoblots.

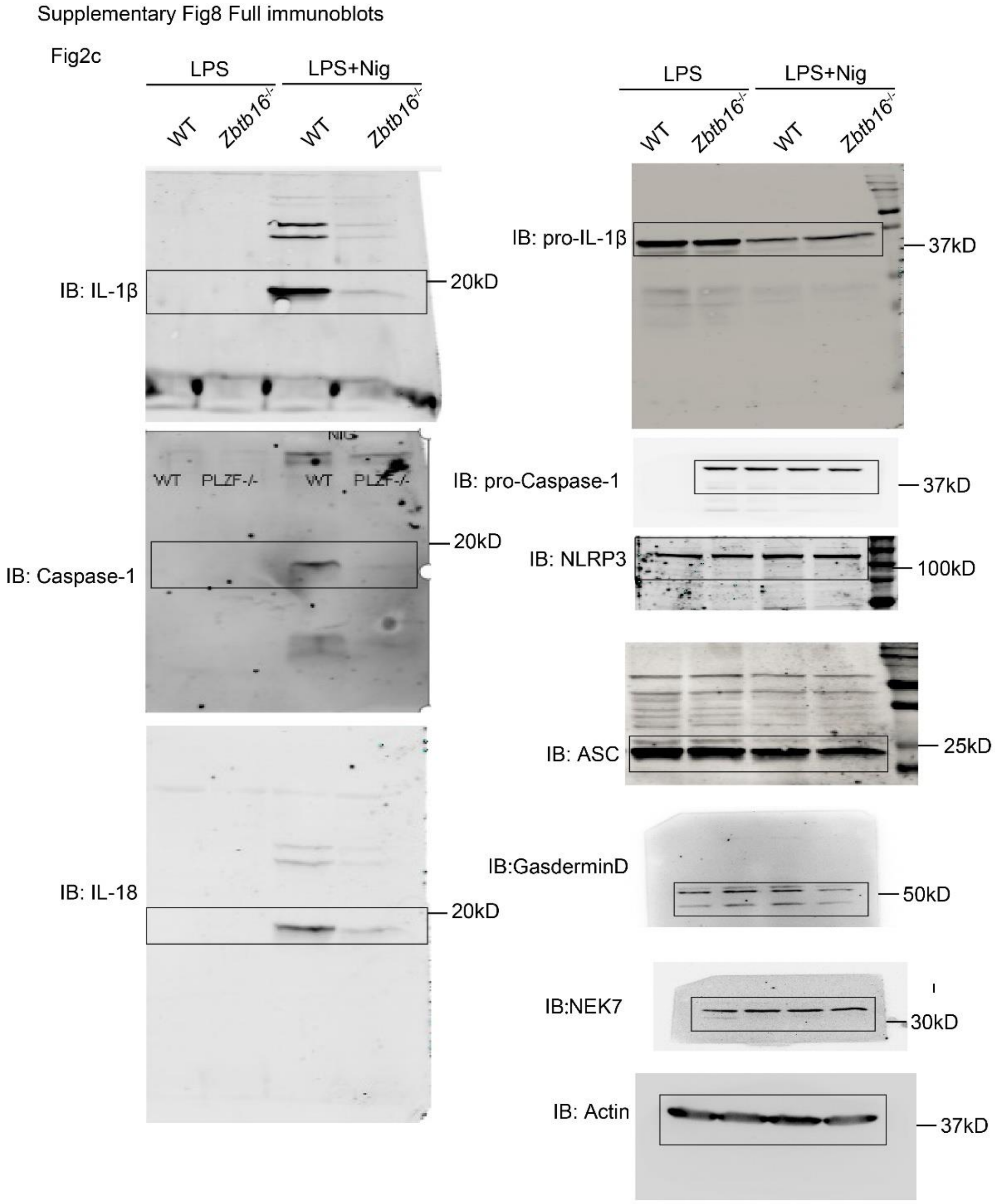

Fig2d

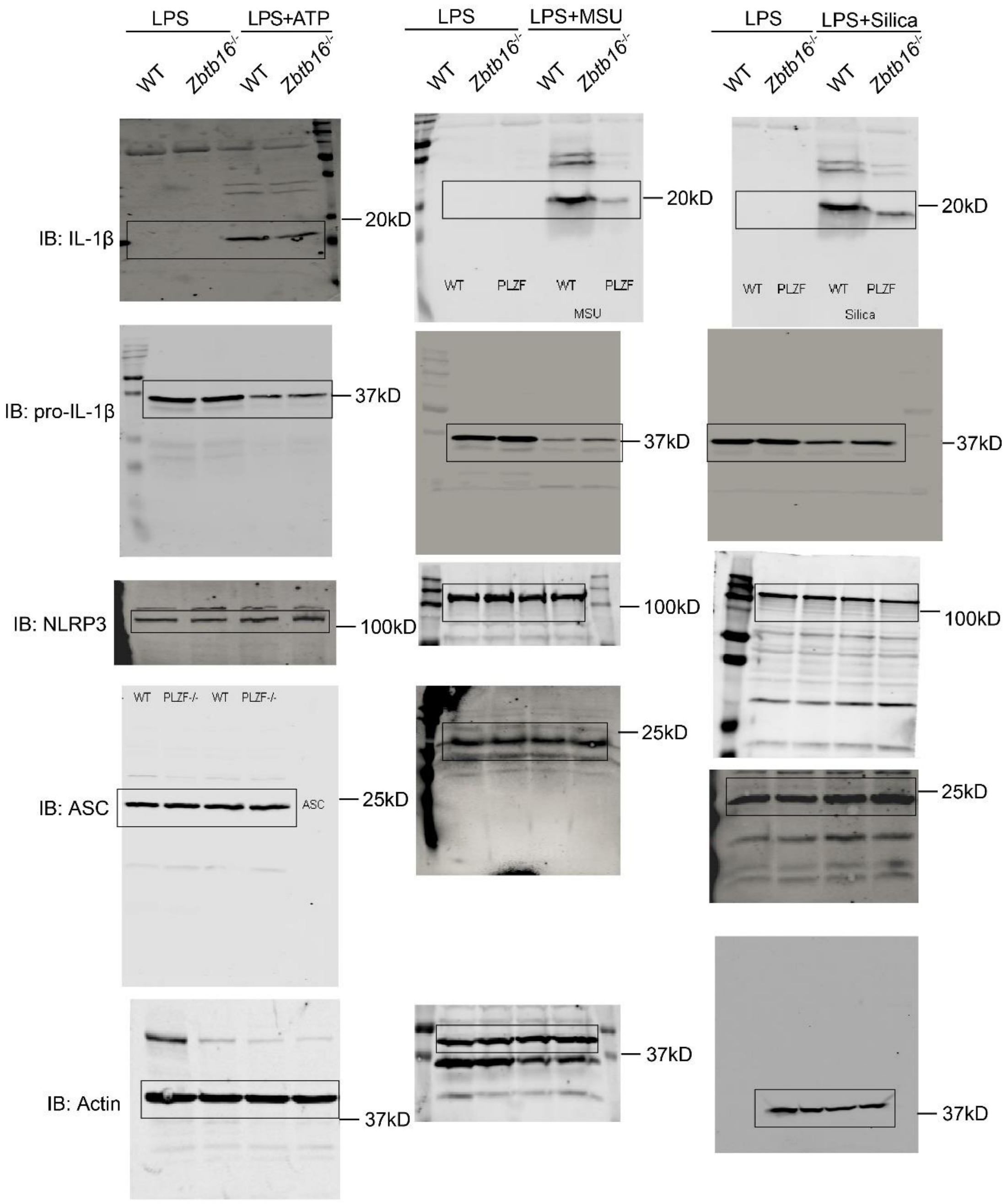

LPS
LPS+ATP
WT
Zbtb16-/-
WT
Zbtb16-/-
LPS
LPS+MSU
WT
Zbtb16-/-
WT
Zbtb16-/-
LPS
LPS+Silica
WT
Zbtb16-/-
WT
Zbtb16-/-
IB: IL-1β
20kD
WT PLZF WT PLZF
MSU
20kD
WT PLZF WT PLZF
Silica
20kD
IB: pro-IL-1β
37kD
37kD
37kD
IB: NLRP3
100kD
100kD
100kD
IB: ASC
WT PLZF-/- WT PLZF-/-
ASC
25kD
25kD
25kD
IB: Actin
37kD
37kD
37kD

Fig4a

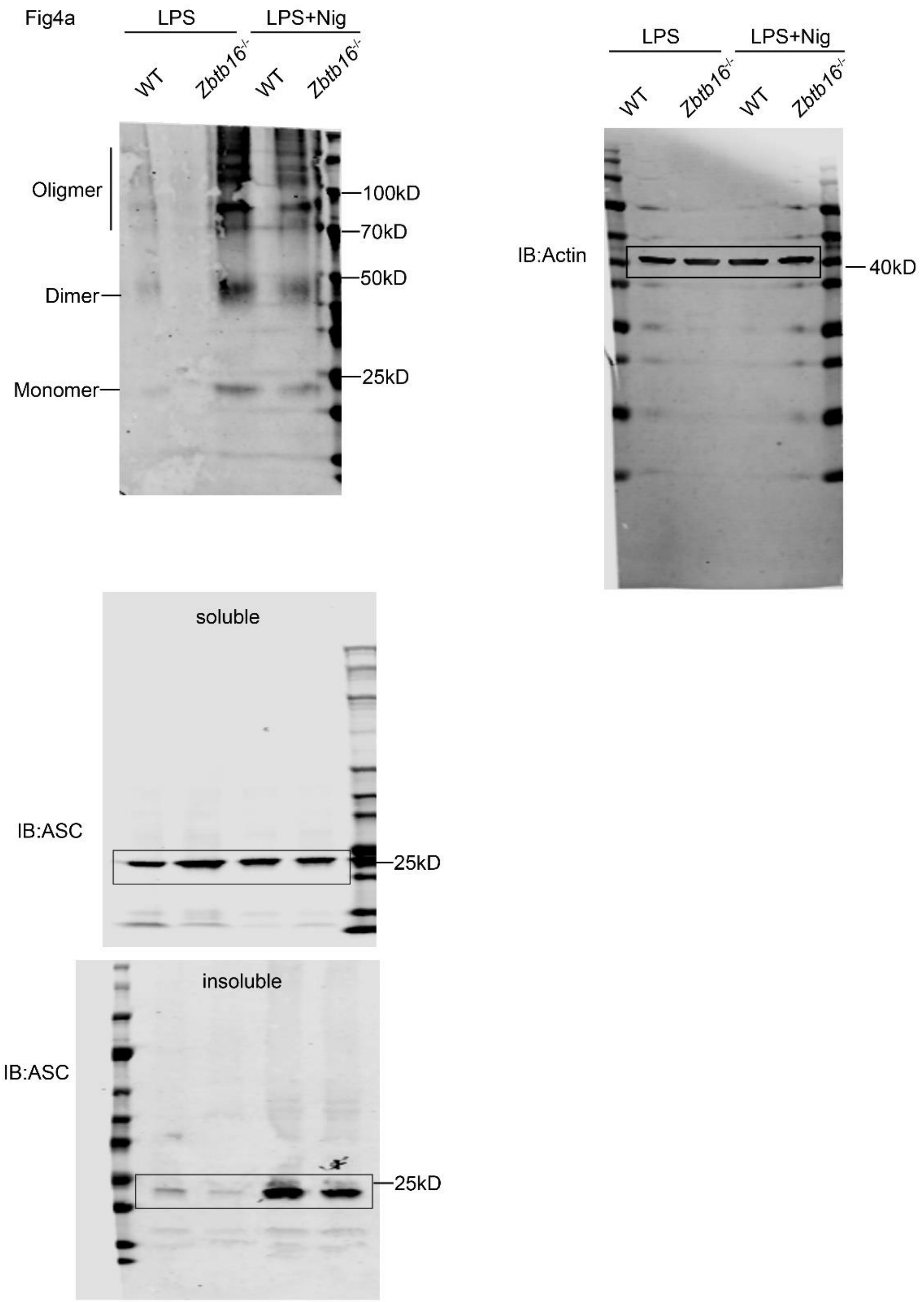

LPS
LPS+Nig
WT
Zbtb16-/-
WT
Zbtb16-/-
Oligmer
Dimer
Monomer
100kD
70kD
50kD
25kD
LPS
LPS+Nig
WT
Zbtb16-/-
WT
Zbtb16-/-
IB:Actin
40kD
soluble
IB:ASC
25kD
insoluble
IB:ASC
25kD

Fig4d

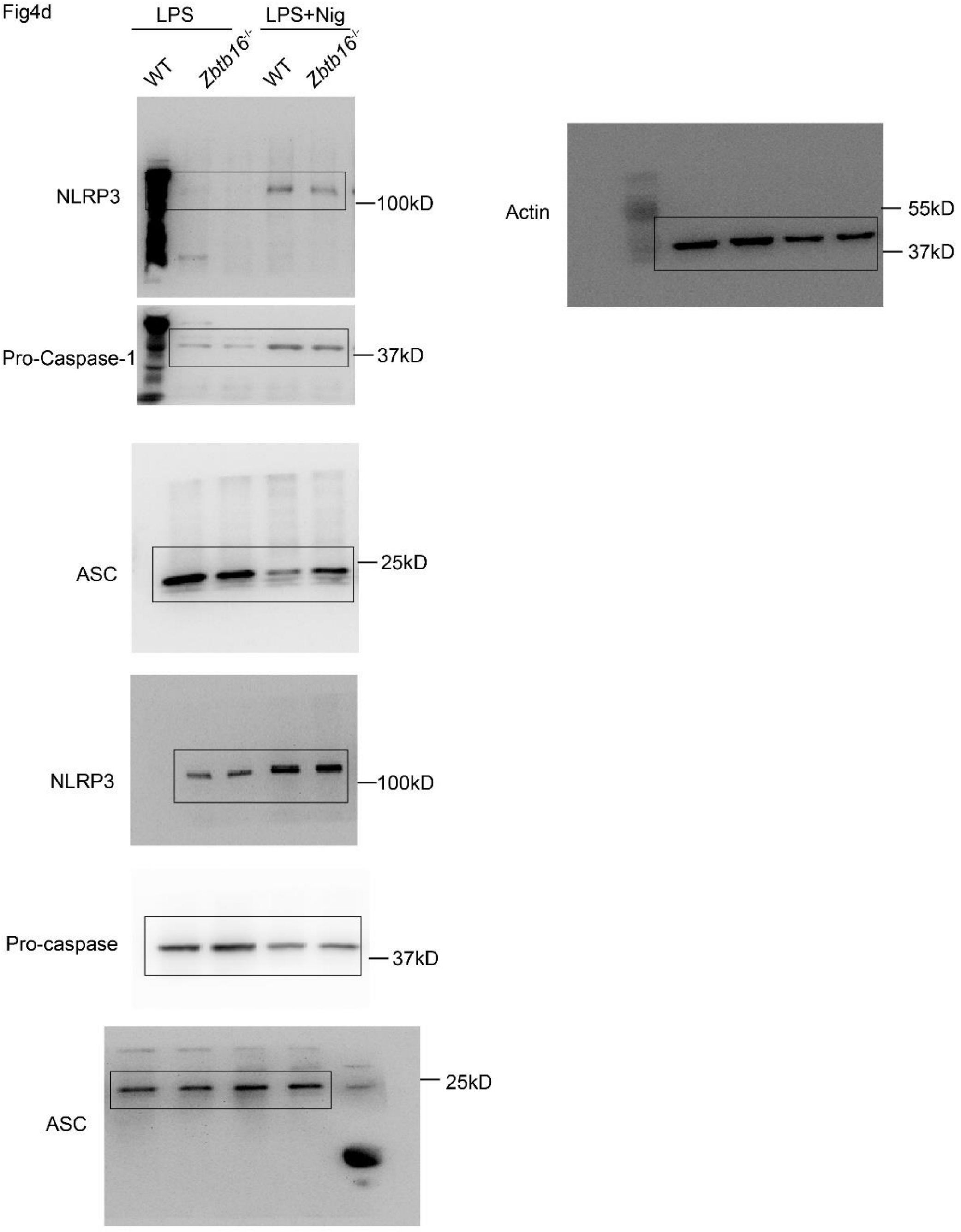

LPS
LPS+Nig
WT
Zbtb16-/-
WT
Zbtb16-/-
NLRP3
100kD
Pro-Caspase-1
37kD
ASC
25kD
NLRP3
100kD
Pro-caspase
37kD
ASC
25kD
Actin
55kD
37kD

Figure5a

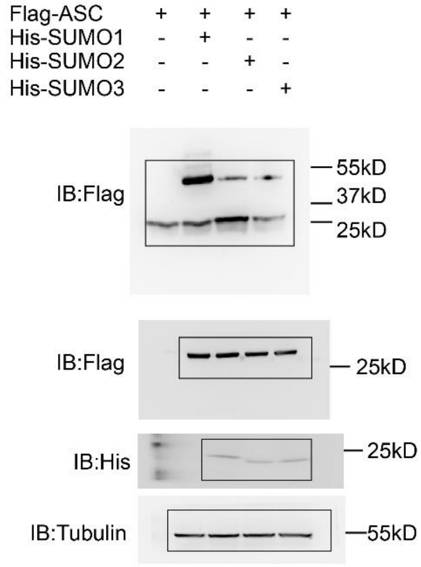

Figure5b

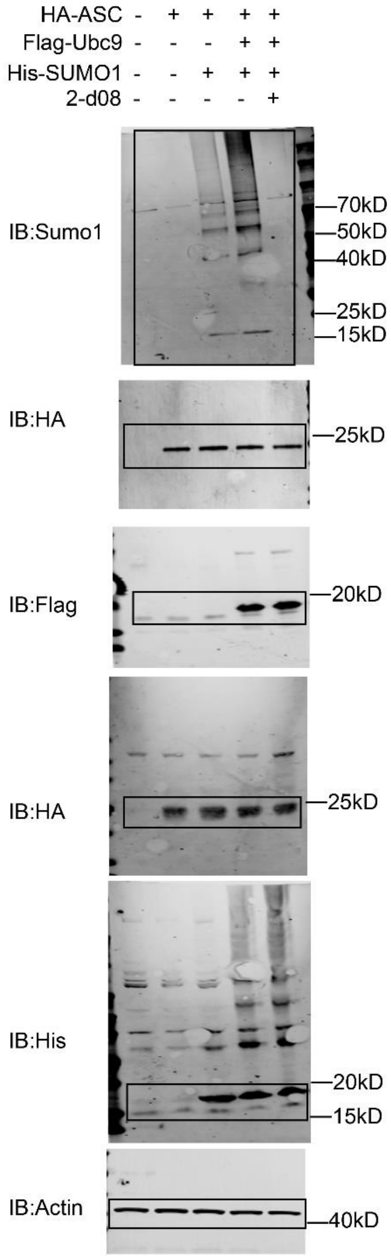

Figure5c

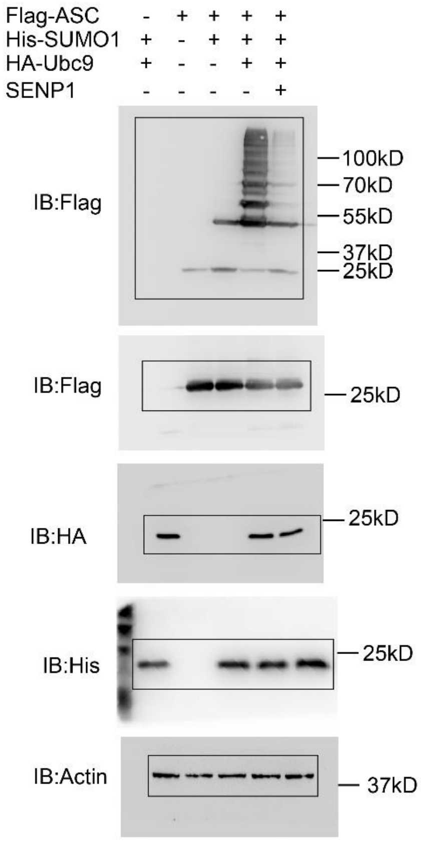

Figure5d

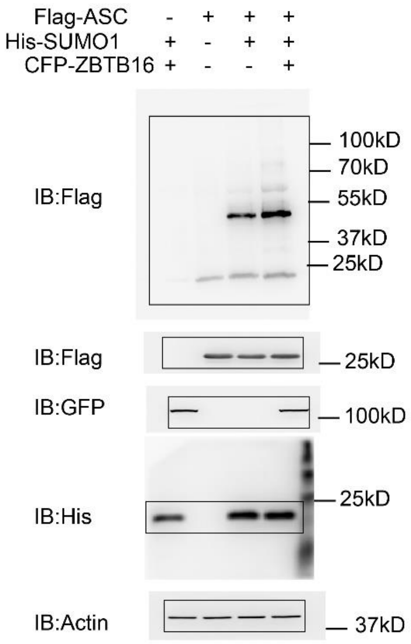

Figure5e

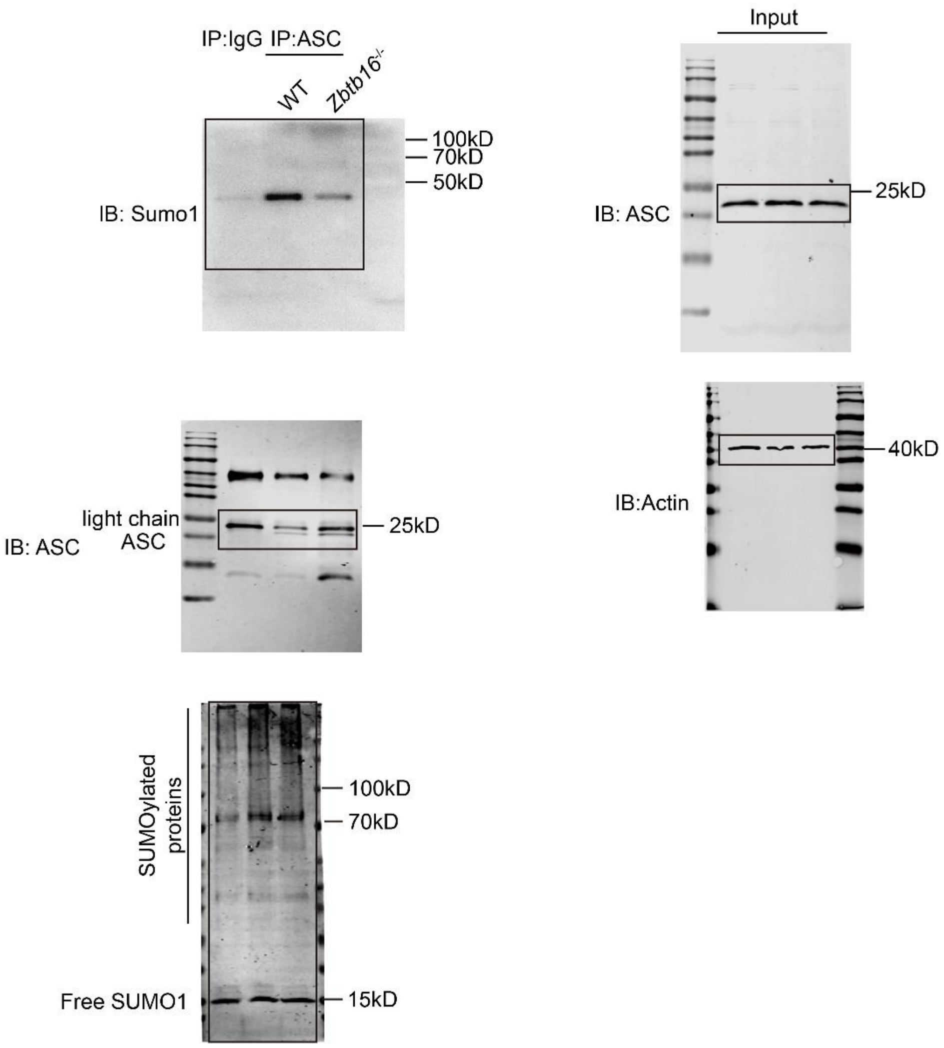
IP:IgG
IP:ASC
WT
Zbtb16-/-
100kD
70kD
50kD
IB: Sumo1
Input
25kD
IB: ASC
light chain
ASC
IB: ASC
25kD
IB:Actin
40kD
SUMOylated proteins
100kD
70kD
Free SUMO1
15kD

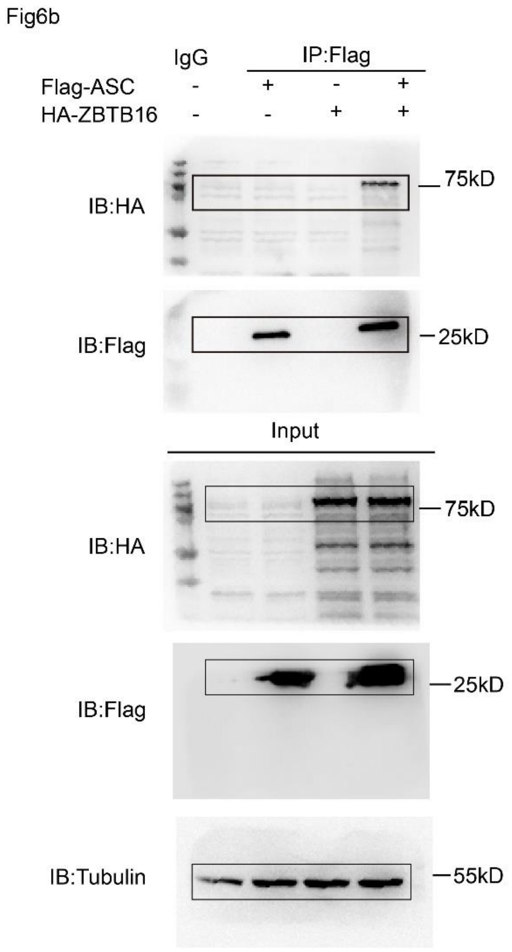
Fig6b
IgG
IP:Flag
Flag-ASC - + - +
HA-ZBTB16 - - + +
IB:HA
75kD
IB:Flag
25kD
Input
IB:HA
75kD
IB:Flag
25kD
IB:Tubulin
55kD

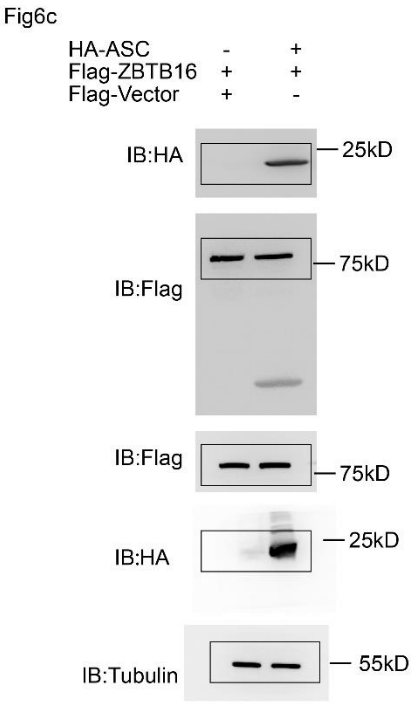
Fig6c
HA-ASC - +
Flag-ZBTB16 + +
Flag-Vector + -
IB:HA
25kD
IB:Flag
75kD
IB:Flag
75kD
IB:HA
25kD
IB:Tubulin
55kD

Figure7a

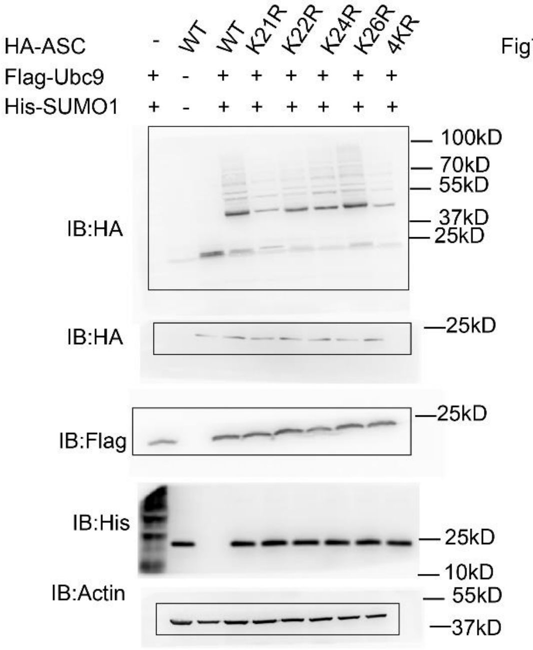

Fig7d

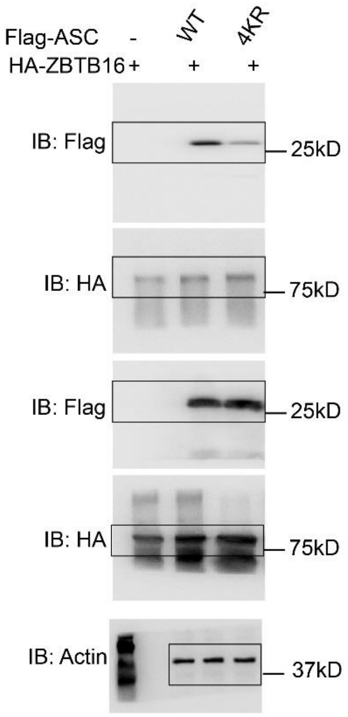

Figure7b

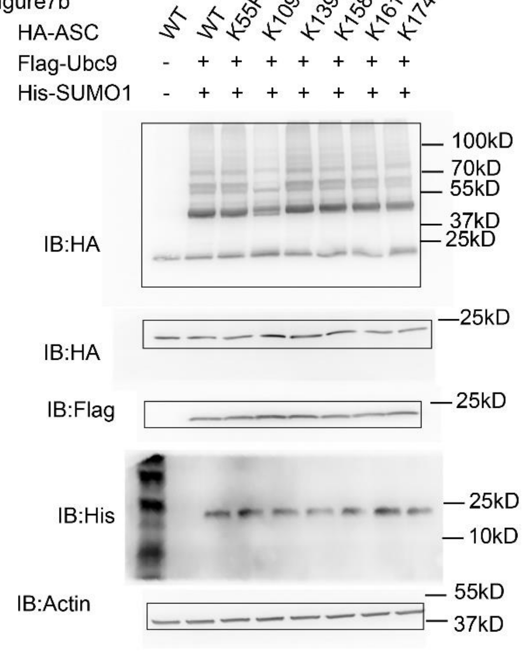

Fig7e

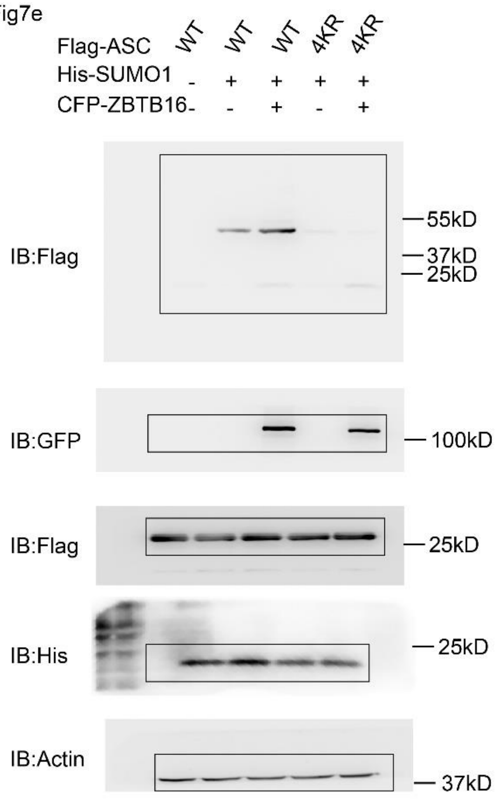
Flag-ASC WT WT WT 4KR 4KR
His-SUMO1 - + + + +
CFP-ZBTB16 - - + - +
IB:Flag
55kD
37kD
25kD
IB:GFP
100kD
IB:Flag
25kD
IB:His
25kD
IB:Actin
37kD

Fig7f

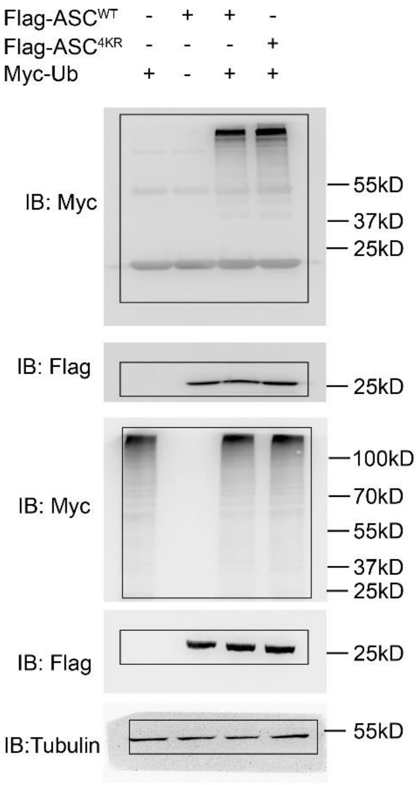
Flag-ASC^WT - + + -
Flag-ASC^4KR - - - +
Myc-Ub + - + +
IB: Myc
55kD
37kD
25kD
IB: Flag
25kD
IB: Myc
100kD
70kD
55kD
37kD
25kD
IB: Flag
25kD
IB:Tubulin
55kD

Fig8a

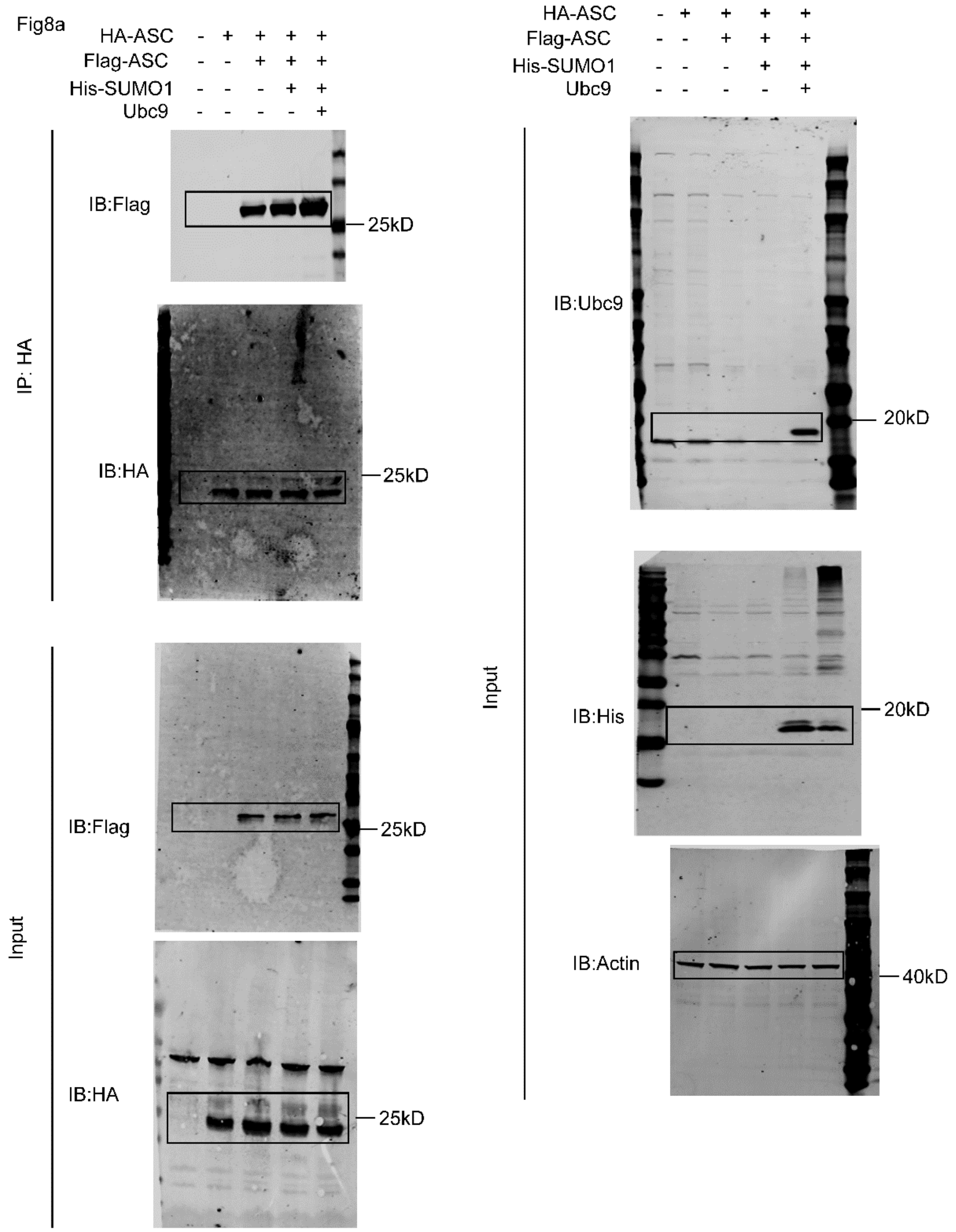
HA-ASC - + + + +
Flag-ASC - - + + +
His-SUMO1 - - - + +
Ubc9 - - - - +
IP: HA
IB:Flag
25kD
IB:HA
25kD
Input
IB:Flag
25kD
IB:HA
25kD
HA-ASC - + + + +
Flag-ASC - - + + +
His-SUMO1 - - - + +
Ubc9 - - - - +
Input
IB:Ubc9
20kD
IB:His
20kD
IB:Actin
40kD

Fig8b

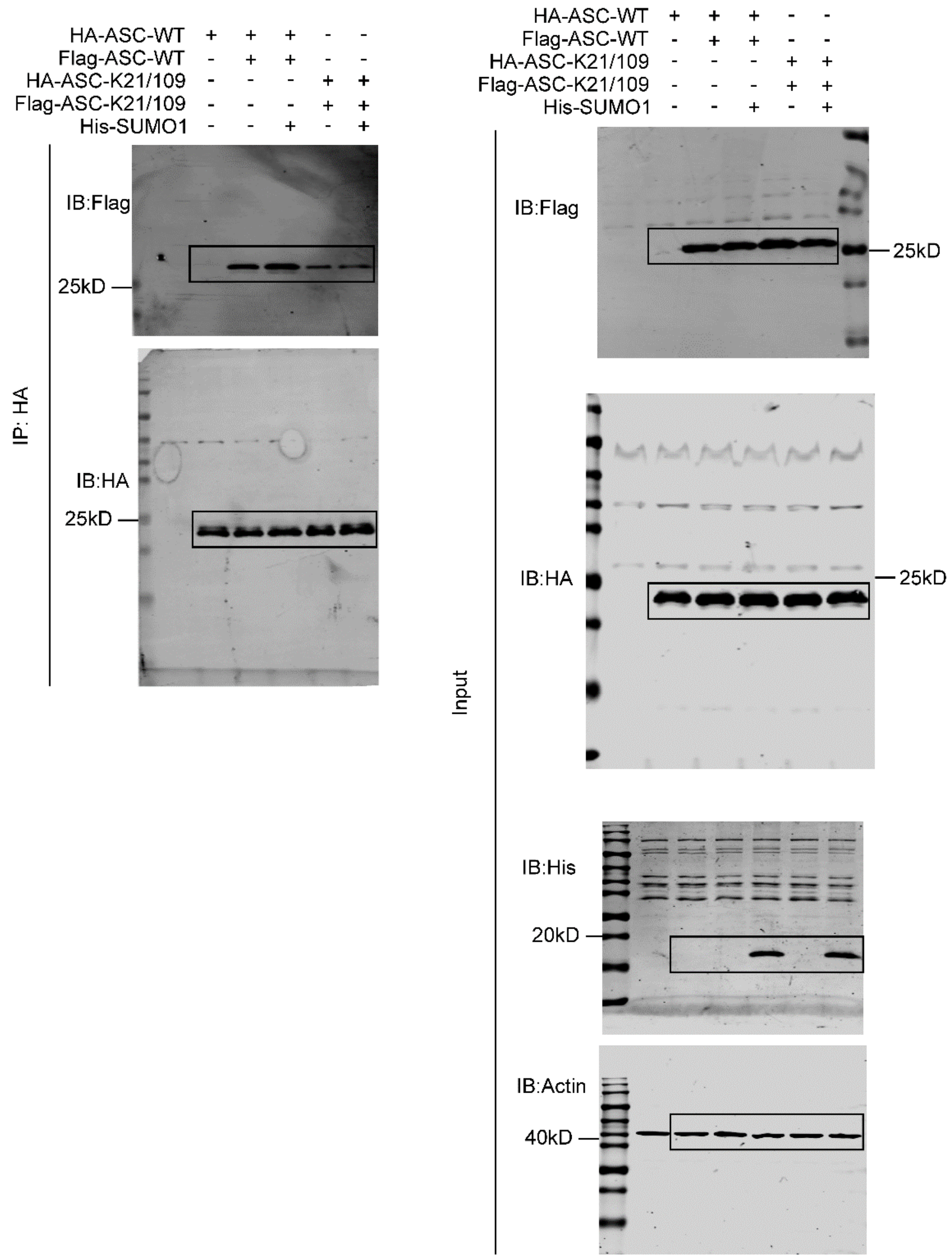

HA-ASC-WT + + + - -
Flag-ASC-WT - + + - -
HA-ASC-K21/109 - - - + +
Flag-ASC-K21/109 - - - + +
His-SUMO1 - - + - +
IP: HA
IB:Flag
25kD
IB:HA
25kD
HA-ASC-WT + + + - -
Flag-ASC-WT - + + - -
HA-ASC-K21/109 - - - + +
Flag-ASC-K21/109 - - - + +
His-SUMO1 - - + - +
Input
IB:Flag
25kD
IB:HA
25kD
IB:His
20kD
IB:Actin
40kD

Supplementary Fig2b

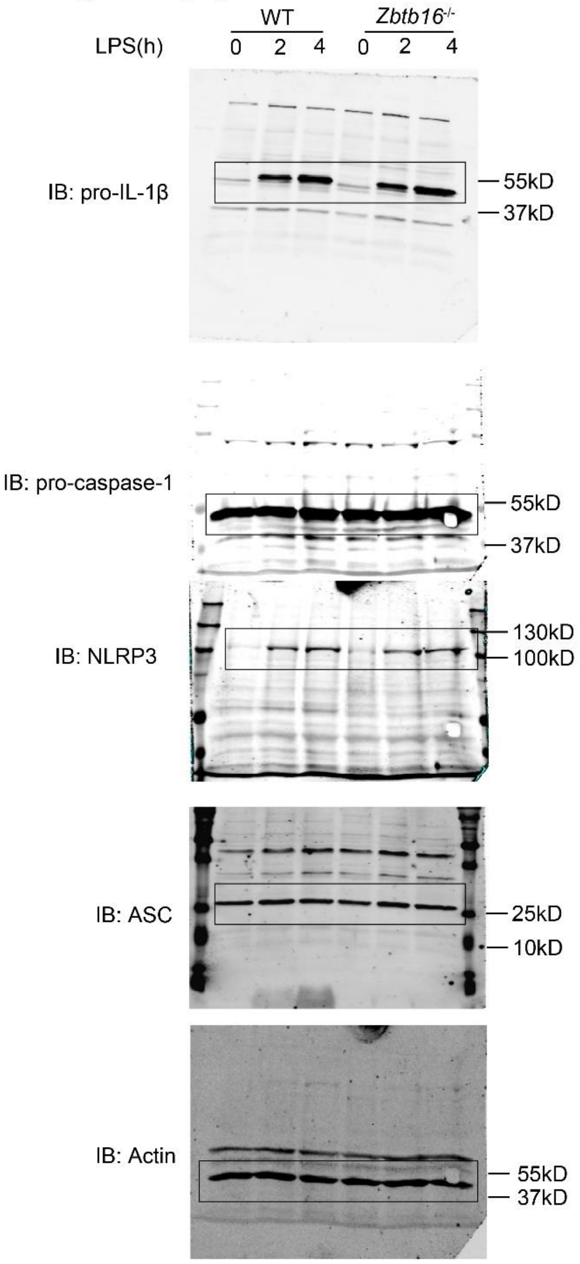

Supplementary Fig5a

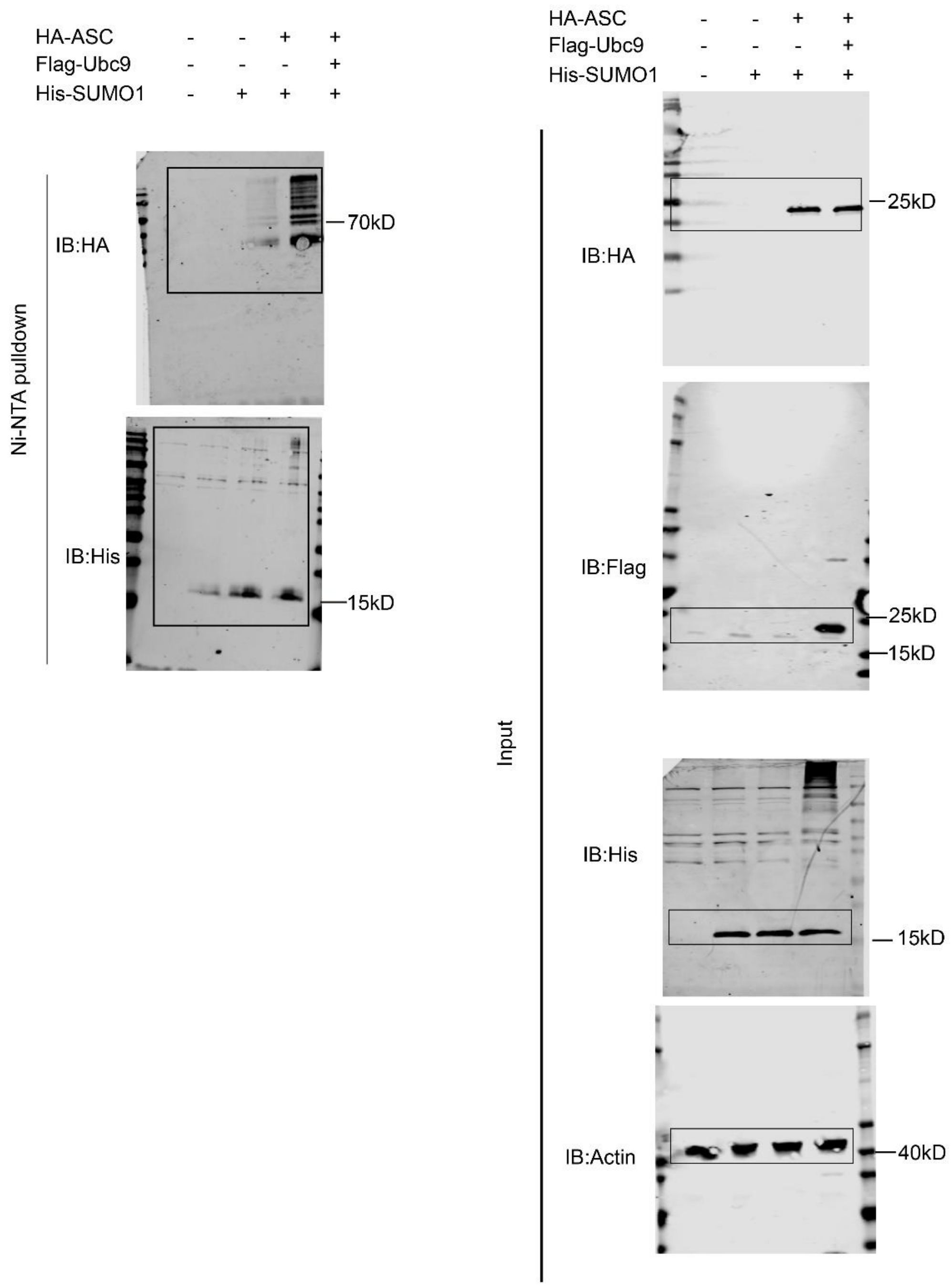

Supplementary Fig5c

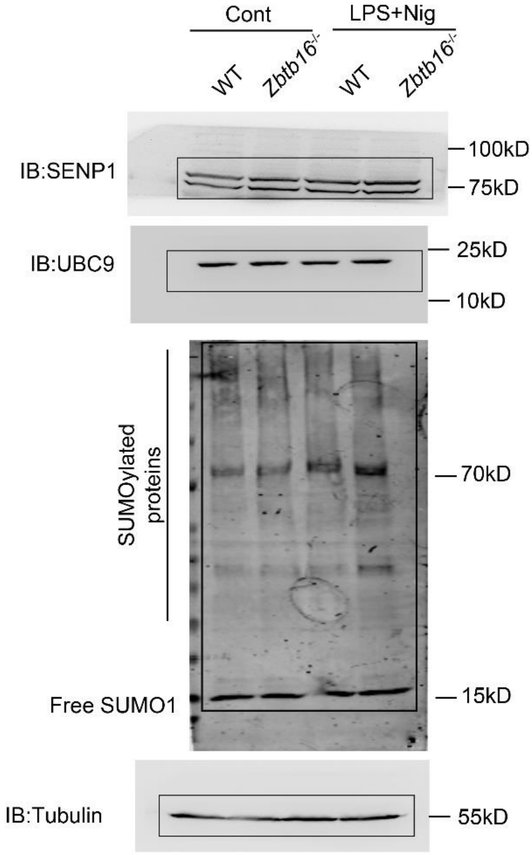

Supplementary Fig7b

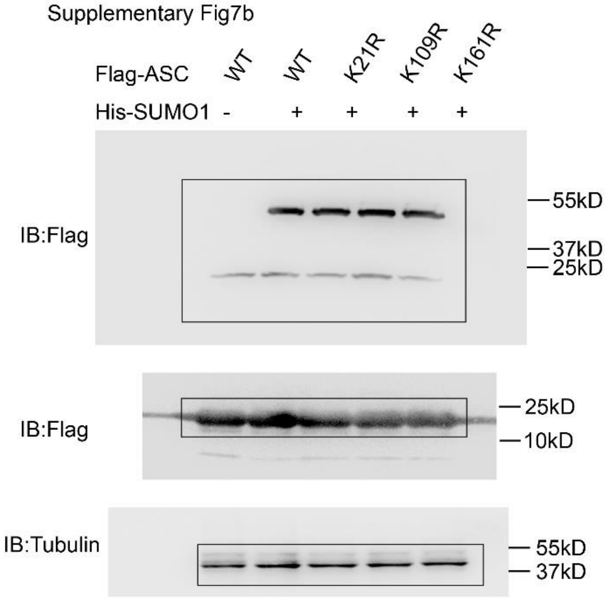

Supplementary Fig7c

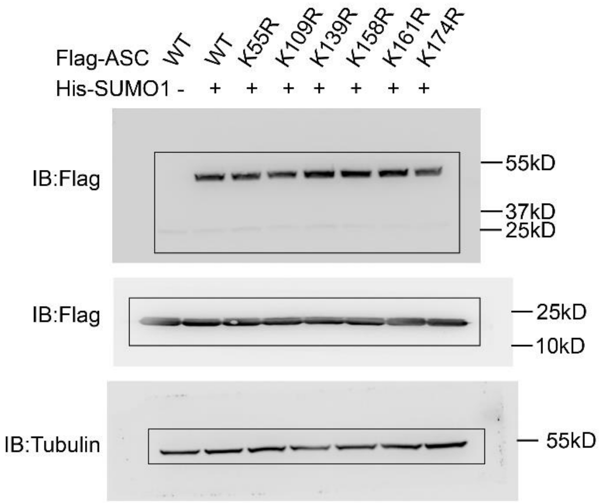

Supplementary Fig7d

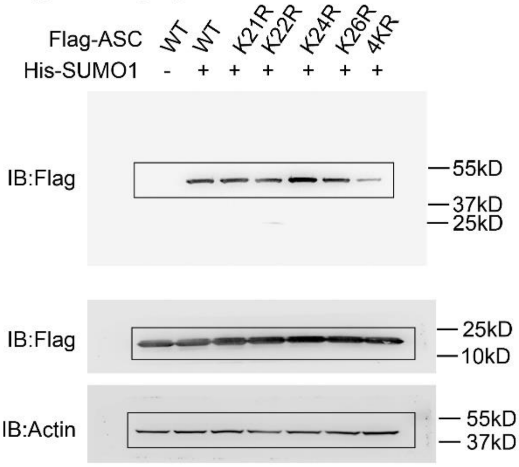

Supplementary Fig7f

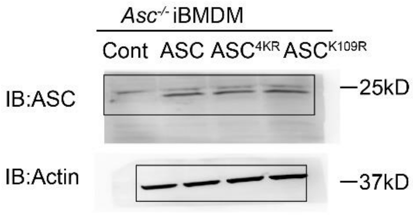

Supplementary Table 1 Primers used for generation of CRISPR/Cas9 inducible knockout cell line.

Primer	Sequence (5’-3’)
zbtb16_gRNA_1F	TCCCTGTGCAAGGCCAACCAGATGCGG
zbtb16_gRNA_1R	AAACCCGCATCTGGTTGGCCTTGCACA
zbtb16_gRNA_2F	TCCCCATCTCGAAGCATTCCAGCGAGG
zbtb16_gRNA_2R	AAACCCTCGCTGGAATGCTTCGAGATG
zbtb16_gRNA_3F	TCCCTGTAGCTGCGCACGCCCGCG
zbtb16_gRNA_3R	AAACCGCGGGCGTGCGCAGCTACA
zbtb16_gRNA_4F	TCCCCGCCACCTGCGCTCACATAC
zbtb16_gRNA_4R	AAACGTATGTGAGCGCAGGTGGCG
zbtb16_RT-PCR_1F	CCTCAGACGACAATGACACGG
zbtb16_RT-PCR_1R	CTCGCTGGAATGCTTCGAGAT

Supplementary Table 2 primers used for construction of ASC mutated vector.

Primer	Sequence (5'-3')
ASC_K21R_F	AGGAAGTTCAAGCTGAAGCTGCTGT
ASC_K21R_R	GAGCTCCTCGGCGGTCAGGTTCTCC
ASC_K22R_F	AAGAGGTTCAAGCTGAAGCTGCTGT
ASC_K22R_R	GAGCTCCTCGGCGGTCAGGTTCTCC
ASC_K24R_F	AAGAAGTTCAGGCTGAAGCTGCTGT
ASC_K24R_R	GAGCTCCTCGGCGGTCAGGTTCTCC
ASC_K26R_F	AAGAAGTTCAAGCTGAGGCTGCTGT
ASC_K26R_R	GAGCTCCTCGGCGGTCAGGTTCTCC
ASC_K55R_F	CGCCTTGGACCTCACCGACCGGCTGGTCAGCTTCTACCTG
ASC_K55R_R	CAGGTAGAAGCTGACCAGCCGGTCGGTGAGGTCCAAGGCG
ASC_K109R_F	CCTCCTCAGTCGGCAGCCCGGCCAGGCCTGCACTTTATA
ASC_K109R_R	TATAAAGTGCAGGCCTGGCCGGGCTGCCGACTGAGGAGG
ASC_K139R_F	AGGGTCCTGACGGATGAGCAGTAC
ASC_K139R_R	CCCGTACAGAGCATCCAGCAGCCA
ASC_K158R_F	AGGATGCGGAAGCTCTTCAGTTTC
ASC_K158_R	GCTTGGGTTGGTGGGCTCGGCCCG
ASC_K161R_F	AGGCTCTTCAGTTTCACACCA
ASC_K161R_R	CCGCATCTTGCTTGGGTTGGT
ASC_K174R_F	AGGGACTTGCTCCTCCAGGCCCTA
ASC_K174R_R	GCAGGTCCAGTTCCAGGCTGGTGT
ASC_K21/K22/K24/K26(4KR)_F	AGGCTGAGGCTGCTGTCGGTGCCG
ASC_K21/K22/K24/K26(4K)_R	GAACCTCCTGAGCTCCTCGGCGGT

Supplementary Table 3 Reagents and Resources

Category	Antibodies	References	Description	Dilution
**Western Blot Antibodies and Reagents**	Anti-IL-1ß	Abcam#ab9722	Rabbit polyclonal	1:1000
	Anti-Caspase-1	Adipogen#AG-20B-0042	Mouse monoclonal	1:1000
	Anti-IL-18	Abcam#ab71495	Rabbit polyclonal	1:1000
	Anti-NLRP3	Adipogen#AG-20B-0014	Mouse monoclonal	1:1000
	Anti-GasderminD	Abcam#ab209845	Rabbit monoclonal	1:1000
	Anti-NEK7	Abcam#ab133514	Rabbit monoclonal	1:1000
	Anti-ASC	CST#67824	Rabbit monoclonal	1:1000
	Anti-ASC	Adipogen#AG-25B-0006	Rabbit polyclonal	1:1000
	Anti-ASC	Santa Cruz#sc-22514-R	Rabbit polyclonal	1:1000
	Anti-Actin	HUABIO#EM21002	Mouse monoclonal	1:10000
	Anti-Tublin	CST#2146	Rabbit polyclonal	1:1000
	Anti-Flag	CST#14793	Rabbit monoclonal	1:1000
	Anti-HA	CST#3724	Rabbit monoclonal	1:1000
	Anti-HA	Proteintech#66006-2	Mouse monoclonal	1:5000
	Anti-Sumo-1	CST#4940	Rabbit monoclonal	1:1000
	Anti-Sumo-1	Proteintech#67559-1	Mouse monoclonal	1:1000
	Anti-SENP1	Abcam#ab236094	Rabbit polyclonal	1:1000
	Anti-His	MBL#PM032	Rabbit polyclonal	1:5000
	Anti-GFP	Invitrogen#MA5-15256	Mouse monoclonal	1:1000
	Anti-mouse IgG, HRP-linked Antibody	CST#7076	Mouse IgG	1:10000
	Anti-rabbit IgG, HRP-linked Antibody	CST#7074	Rabbit IgG	1:10000
	Multicolor Prestained Protein Ladder	Epizyme#WJ106		
	IRDye 680RD Goat anti-Rabbit IgG Secondary Antibody	LI-COR#925-68071		1:20000
	IRDye 800CW Goat anti-Mouse IgG	LI-COR#925-32210		1:20000

	Secondary Antibody			
	Intercept (TBS) Blocking Buffer	LI-COR#927-60001		
**Immunoprecipitation Antibodies and Reagents**	Anti-ASC	Adipogen#AG-25B-0006	Rabbit polyclonal	1:100
	Anti-ASC	Biolegend#653902	Mouse monoclonal	1:100
	Anti-HA	CST#3724	Rabbit monoclonal	1:100
	Anti-Flag	CST#14793	Rabbit monoclonal	1:100
	Anti-Sumo-1	CST#4940	Rabbit monoclonal	1:100
	VeriBlot for IP Detection Reagent (HRP)	Abcam#ab131366		1:1000
	Protein A+G agarose (Fast Flow for IP)	Beyotime Biotechnology#P2055		-
	HA Nanoab Mag Beads	EveryLab#IP07M		
	3xflag peptide	EveryLab#PF03		
	Anti-DYKDDDDK G1 Affinity Resin	GenScript#L00432		
	Ni-NTA Agarose	QIAGEN# 30210		-
	Pierce Co-Immunoprecipitation Kit from Thermo Fisher	Thermo Fisher #26149		
**Immunofluorescence/IHC Antibodies**	Anti-S100A9	CST#72590	Rabbit monoclonal	1:100
	Anti-Ly6G	Bio X Cell BP0075-1	Rat monoclonal	1:100
	Anti-ASC	Santa Cruze#sc-271054	Mouse monoclonal	1:100
	Anti-ASC	Biolegend #653902	Mouse monoclonal	1:100
	Anti-PML	Santa Cruz#sc-377390	Mouse monoclonal	1:100
	Anti-Ubc9	Abcam#ab75854	Rabbit monoclonal	1:100
	Anti-Sumo-1	CST#4930	Rabbit polyclonal	1:100
	Anti-PLZF	Calbiochem#OP128	Mouse monoclonal	1:100
	DAPI	CST#4083	-	1:20000
	Alexa 488 anti-rabbit	Invitrogen#A32731	-	1:100
	Alexa 594 anti-rabbit	Invitrogen#A32740	-	1:100

	Alexa 488 anti-mouse	Invitrogen#A32723	-	1:100
	Alexa 555 anti-mouse	Invitrogen#A32727	-	1:100
**PLA antibodies and Reagents**	Anti-ASC	Adipogen#AG-25B-006	Rabbit polyclonal	1:200
	Anti-ASC	Santa Cruze#sc-271054	Mouse monoclonal	1:50
	Anti-PLZF	Bioss#bs-5971R	Mouse polyclonal	1:100
	Anti-Sumo-1	CST#4930	Rabbit polyclonal	1:200
	Anti-NLRP3	Adipogen#AG-20B-0014	Mouse monoclonal	1:200
	Duolink(R) In Situ Detection Reagents Red	Sigma#DUO92008		
	Duolink(R) In Situ PLA(R) Probe Anti-Rabbit PLUS	Sigma#DUO92002		
	Duolink(R) In Situ Wash Buffers, Fluorescence	Sigma#DUO82049		
	Duolink(R) In Situ PLA(R) Probe Anti-Mouse MINUS	Sigma#DUO92004		
**Flow Cytometer Antibodies**	APC anti-mouse CD45	Biolegend#103112	-	-
	PE anti-mouse Ly-6G	Biolegend#127608	-	-
	PerCP/Cyanine5.5 anti-mouse/human CD11b	Biolegend#101228	-	-
	Zombie NIR Fixable Viability Kit	Biolegend#423106	-	-
**Chemicals**	Monosodium Urate Crystals (MSU)	invivogene#tlrl-msu	-	-
	Adenosine-triphosphate disodium salt (ATP)	invivogene#tlrl-atpl	-	-
	Nano-SiO2(Silica)	invivogene#tlrl-sio	-	-
	Nigericin, sodium salt	invivogene#tlrl-nig	-	-
	Toxin B from Clostridium difficile	SantaCruz#sc-222362	-	-
	Lipopolysaccharides from Escherichia coli O111:B4	SIGMA#L3012	-	-
	C.difficile	ATCC#BAA-1382	-	-
	Doxycycline hyclate	SIGMA#D9891	-	-
	2-D08	Selleck# S8696		

	DSS	ThermoFisher#21655		
	EZ-Trans cell Transfection Reagent	Life-iLab#AC04L092		
	Recombinant Mouse GM-CSF (C-6His)	Novoprotein#CJ46		
**Critical Commercial Kits**	ELISA MAX Deluxe Set Mouse IL-1ß	Biolegend#432604	-	-
	IL-18 Mouse ELISA Kit	Thermo Fisher(eBioscience)#BMS618-3	-	-
	ELISA MAX Deluxe Set Mouse TNFa	Biolegend#430915	-	-
	LDH release assay	Beyotime Biotechnology#C0016	-	-
	Myeloperoxidase(MPO) Peroxidation Activity Assay Kit	Biovision #K747-100		
	PAGE Gel Fast Preparation Kit （12.5%）	Epizyme#PG113		
	YoungPage,Bis-Tris,4-20%,11wells	GenScript#M00928		

Supplementary Table 4 primers used for RT-PCR

Primer	Sequence (5’-3’)
mouse Asc_F	CAGAGTACAGCCAGAACAGGACAC
mouse Asc_R	GTGGTCTCTGCACGAACTGCCTG
mouse Nlrp3_F	CGAGACCTCTGGGAAAAAGCT
mouse Nlrp3_R	GCATACCATAGAGGAATGTGATGTACA
mouse Casp1_F	TCCGCGGTTGAATCCTTTTCAGA
mouse Casp1_R	ACCACAATTGCTGTGTGTGCGCA
mouse Il1ß_F	TCTGGGATCCTCTCCAGCCAAG
mouse Il1ß_R	TCAGGACAGCCCAGGTCAAAGG
mouse Il18_F	CAGTGAACCCCAGACCAGAC
mouse Il18_R	TGTTGTGTCCTGGAACACGT
