# Supplementary material for: Inflammasome activity is controlled by ZBTB16-dependent SUMOylation of ASC
Source: Nat Commun. 2023 Dec 20;14:8465. doi: 10.1038/s41467-023-43945-1 (PMC10733316; doi:10.1038/s41467-023-43945-1)
Supplement: Supplementary file 3 — Reporting Summary [file 41467_2023_43945_MOESM3_ESM.pdf]

nature portfolio

Corresponding author(s): Dakang Xu, Anthony Sadler and Yibing Peng

Last updated by author(s): Oct 15, 2023

Reporting Summary

Nature Portfolio wishes to improve the reproducibility of the work that we publish. This form provides structure for consistency and transparency in reporting. For further information on Nature Portfolio policies, see our Editorial Policies and the Editorial Policy Checklist.

Statistics

For all statistical analyses, confirm that the following items are present in the figure legend, table legend, main text, or Methods section.

n/a	Confirmed	
☐	☒	The exact sample size (*n*) for each experimental group/condition, given as a discrete number and unit of measurement
☐	☒	A statement on whether measurements were taken from distinct samples or whether the same sample was measured repeatedly
☐	☒	The statistical test(s) used AND whether they are one- or two-sided<br>*Only common tests should be described solely by name; describe more complex techniques in the Methods section.*
☒	☐	A description of all covariates tested
☒	☐	A description of any assumptions or corrections, such as tests of normality and adjustment for multiple comparisons
☐	☒	A full description of the statistical parameters including central tendency (e.g. means) or other basic estimates (e.g. regression coefficient) AND variation (e.g. standard deviation) or associated estimates of uncertainty (e.g. confidence intervals)
☐	☒	For null hypothesis testing, the test statistic (e.g. *F*, *t*, *r*) with confidence intervals, effect sizes, degrees of freedom and *P* value noted<br>*Give P values as exact values whenever suitable.*
☒	☐	For Bayesian analysis, information on the choice of priors and Markov chain Monte Carlo settings
☒	☐	For hierarchical and complex designs, identification of the appropriate level for tests and full reporting of outcomes
☒	☐	Estimates of effect sizes (e.g. Cohen's *d*, Pearson's *r*), indicating how they were calculated

Our web collection on statistics for biologists contains articles on many of the points above.

Software and code

Policy information about availability of computer code

Data collection	Applied Biosystems 7700 Prism real-time PCR machine; Zeiss LSM710 confocal fluorescence microscope with objective Plan-Apochromat 63×/1.40 oil DIC M27 objective; FACS data was collected by FACSCantoII (BD Biosciences);Odyssey Imaging System (LI-COR, USA); Olympus U-RFL-T burner, BX60 microscope using a DP74 camera and the Olympus CellSens software; Nikon DS-F2 microscope; the Aperio ScanScope XT imaging system (Aperio, Vista) software.
Data analysis	Statistics were analyzed by GraphPad Prism 8.0. Quantification of Western Blot bands were done by Image J.<br>The FACS data was analyzed by Flowjo v10.<br>The fluorescent signal was analysed using the ImageJ software (NIH and LOCI), Colocalization score was calculated using the GcoPS tool (http://icy.bioimageanalysis.org/plugin/GcoPS).<br>The overlap between the 3D surface-reconstructed images was processed using the IMARIS surface-surface overlap module.

For manuscripts utilizing custom algorithms or software that are central to the research but not yet described in published literature, software must be made available to editors and reviewers. We strongly encourage code deposition in a community repository (e.g. GitHub). See the Nature Portfolio guidelines for submitting code & software for further information.

Data

Policy information about availability of data

All manuscripts must include a data availability statement. This statement should provide the following information, where applicable:

- Accession codes, unique identifiers, or web links for publicly available datasets
- A description of any restrictions on data availability
- For clinical datasets or third party data, please ensure that the statement adheres to our policy

All data supporting the findings of this study are included in the text, figures, and supplementary materials.

Research involving human participants, their data, or biological material

Policy information about studies with human participants or human data. See also policy information about sex, gender (identity/presentation), and sexual orientation and race, ethnicity and racism.

Reporting on sex and gender	Not involved
Reporting on race, ethnicity, or other socially relevant groupings	Not involved
Population characteristics	Not involved
Recruitment	Not involved
Ethics oversight	Not involved

Note that full information on the approval of the study protocol must also be provided in the manuscript.

Field-specific reporting

Please select the one below that is the best fit for your research. If you are not sure, read the appropriate sections before making your selection.

[x] Life sciences [ ] Behavioural & social sciences [ ] Ecological, evolutionary & environmental sciences

For a reference copy of the document with all sections, see nature.com/documents/nr-reporting-summary-flat.pdf

Life sciences study design

All studies must disclose on these points even when the disclosure is negative.

Sample size	The numbers of samples used needed to be sufficient to result in statistically significant differences using standard power calculations.
Data exclusions	No data was excluded.
Replication	All experiments were carried out at least three times, and the attempts to replicate the results were successful.
Randomization	For in vitro studies, the samples/cells were randomized into different groups prior to treatment. For animal experiments, all mice were randomly divided into each group before receiving different treatment.
Blinding	Data collection and/or analysis in this study were conducted in a blind manner.

Reporting for specific materials, systems and methods

We require information from authors about some types of materials, experimental systems and methods used in many studies. Here, indicate whether each material, system or method listed is relevant to your study. If you are not sure if a list item applies to your research, read the appropriate section before selecting a response.

Materials & experimental systems

n/a	Involved in the study
☐	☒ Antibodies
☐	☒ Eukaryotic cell lines
☒	☐ Palaeontology and archaeology
☐	☒ Animals and other organisms
☒	☐ Clinical data
☒	☐ Dual use research of concern
☒	☐ Plants

Methods

n/a	Involved in the study
☒	☐ ChIP-seq
☐	☒ Flow cytometry
☒	☐ MRI-based neuroimaging

Antibodies

Antibodies used

Anti-IL-1ß（Abcam#ab9722,1:1000），Anti-Caspase-1（Adipogen#AG-20B-0042,1:1000）,Anti-IL-18 (Abcam #ab71495,1:1000), Western Blot:Anti-NLRP3 (Adipogen #AG-20B-0014,1:1000) , Anti-GasderminD (Abcam#ab209845,1:1000), Anti-NEK7 (Abcam#ab133514,1:1000) ,Anti-ASC (CST#67824,1:1000) , Anti-ASC (Santa Cruz#sc-22514-R,1:1000), Anti-Actin (Proteintech#60008,1:10000), Anti-Tubulin (CST#2146,1:1000), Anti-Flag (CST#14793,1:1000), Anti-HA (CST#3724,1:1000) Anti-Sumo1 (CST#4940,1:1000), Anti-SENP1 (Abcam#ab236094,1:1000), Anti-His (MBL#PM032, 1:5000),Anti-GFP (Invitrogen#MA5-15256,1:1000), Anti-mouse IgG (HRP-linkedAntibody CST#7076,1:10000) ,Anti-rabbit IgG (HRP-linked Antibody CST#7074,1:10000) ,IRDye® 680RD Goat anti-Rabbit IgG Secondary Antibody(LI-COR#925-68071,1:20000),IRDye® 800CW Goat anti-Mouse IgG Secondary Antibody(LI-COR#925-32210,1:20000)

Immunoprecipitation: Anti-ASC (Adipogen#AG-25B-0006,1:100),Anti-ASC(Biolegend#653902,1:100), Anti-HA(CST#3724,1:100),Anti-Flag(CST#14793,1:100),Anti-Sumo-1(CST#4940,1:100),VeriBlot for IP Detection Reagent (HRP)(Abcam#ab131366,1:1000)

Immunofluorescen/IHC: Anti-S100A9 (CST#72590, 1:100) ,Anti-Ly6G (Bio X Cell BP0075-1,1:100), Anti-ASC (Santa Cruze#sc-271054,1:100),Anti-ASC(Biolegend #653902,1:100),Anti-PML (Santa Cruz#sc-377390,1:100), Anti-Ubc9 (Abcam#ab75854,1:100),Anti-Sumo1 (CST#4930,1:100), Anti-PLZF (Calbiochem#OP128,1:100),DAPI(CST#4083,1:20000),Alexa 488 anti-rabbit(Invitrogen#A32731,1:100),Alexa 594 anti-rabbit(Invitrogen#A32740,1:100),Alexa 488 anti-mouse (Invitrogen#A32723,1:100),Alexa 555 anti-mouse(Invitrogen#A32727,1:100)

PLA:Anti-ASC(Adipogen#AG-25B-006,1:200),Anti-ASC(Santa Cruze#sc-271054,1:50),Anti-PLZF(Bioss#bs-5971R,1:100),Anti-Sumo-1 (CST#4930,1:200),Anti-NLRP3(Adipogen#AG-20B-0014,1:200)

Flow Cytometer: APC anti-mouse CD45 (Biolegend#103112) ,PE anti-mouse Ly-6G (Biolegend#127608),PerCP/Cyanine5.5 antimouse/human CD11b (Biolegend#101228)

Validation

All antibodies are from commercially available sources and have been validated by the supplier for the indicated species and application utilized in our study. Manufacturers' websites contains validation and publications supporting the antibodies use for each species and assay employed.

Eukaryotic cell lines

Policy information about cell lines and Sex and Gender in Research

Cell line source(s)

HEK293T ,Hela, Thp1 were purchased from the ATCC (American Type Culture Collection). ASC-/- BMDM cells were obtained from Eicke Latz (Institute of Innate Immunity, University Hospital Bonn, University of Bonn)

Authentication

The cell lines were not authenticated. All cell lines were kept at low passages in order to maintain their identity.

Mycoplasma contamination

All cell lines were tested mycoplasma free.

Commonly misidentified lines
(See ICLAC register)

All cell lines used in this study are not commonly misidentified lines.

Animals and other research organisms

Policy information about studies involving animals; ARRIVE guidelines recommended for reporting animal research, and Sex and Gender in Research

Laboratory animals

All experiments were performed with mice on the C57BL/6 background aged 6-8week both male and female. Zbtb16-/- mice were reported by our laboratory previously. Zbtb16 conditional knockout mice were generated by BIOCYTOGEN (Beijing, China). Lysozyme M-Cre knock-in mice (CreL) were obtained from the Jackson Laboratories. Nlrp3R258W (+/R258W) mice and Asc-/- mice were reported previously.All mice were bred and maintained under a 12-h reverse light/dark cycle and specific pathogen free (SPF) conditions. The animal facility was maintained at a temperature of 22 ± 2 °C with 40-70 % humidity.

Wild animals

No wild animals were used in this study.

Reporting on sex

Our study involved both male and female mice.

Field-collected samples

No field-collected samples were involved in this study.

Ethics oversight	The animal experiments were approved by the Ethics Committee of Hangzhou Normal University and Ruijin Hospital, Shanghai Jiao Tong University School of Medicine.

Note that full information on the approval of the study protocol must also be provided in the manuscript.

Plants

Seed stocks	Not involved
Novel plant genotypes	Not involved
Authentication	Not involved

Flow Cytometry

Plots

Confirm that:

- [x] The axis labels state the marker and fluorochrome used (e.g. CD4-FITC).
- [x] The axis scales are clearly visible. Include numbers along axes only for bottom left plot of group (a 'group' is an analysis of identical markers).
- [x] All plots are contour plots with outliers or pseudocolor plots.
- [x] A numerical value for number of cells or percentage (with statistics) is provided.

Methodology

Sample preparation	Cells were harvested,resuspended in FACS buffer and then subjected to cytometer.
Instrument	FACSCanto II (BD Biosciences)
Software	FlowJo V10
Cell population abundance	A minimum of 10,000 cells per sample were analyzed
Gating strategy	The gating strategy (FSC-A vs SSC-A)was used to exclude cell debris and aggregates. The singlet population in APC positive channel was gated, and then subjected for further gating of PE and percp-cy5.5 channel.

- [x] Tick this box to confirm that a figure exemplifying the gating strategy is provided in the Supplementary Information.
